# Real-world effectiveness of thrombectomy for basilar artery occlusion: lessons beyond the ATTENTION and BAOCHE trials

**DOI:** 10.1093/esj/aakag031

**Published:** 2026-05-13

**Authors:** Jeong-Yoon Lee, Soo-Hyun Park, Kyungbok Lee, Soo Joo Lee, Kyusik Kang, Jae-Kwan Cha, Hyung Seok Guk, Nakhoon Kim, Jonguk Kim, Do Yeon Kim, Jun Yup Kim, Jihoon Kang, Beom Joon Kim, Moon-Ku Han, Jin-Kyo Choi, Tai Hwan Park, Doo Hyuk Kwon, Jun Lee, Hong-Kyun Park, Yong-Jin Cho, Keun-Sik Hong, Sangwon Choi, Minwoo Lee, Mi Sun Oh, Kyung-Ho Yu, Byung-Chul Lee, Hyunsoo Kim, Kang-Ho Choi, Joon-Tae Kim, Dong-Seok Gwak, Dong-Eog Kim, Joong-Goo Kim, Jay Chol Choi, Kyu Sun Yum, Dong-Ick Shin, Wook-Joo Kim, Jee-Hyun Kwon, Hyung Jong Park, Jeong-Ho Hong, Sung-Il Sohn, Sang-Hwa Lee, Chulho Kim, Chanyoung Park, Hae-Bong Jeong, Kwang-Yeol Park, Jung Hoon Han, Dongwhane Lee, Jong-Moo Park, Byeong-Su Park, Keon-Joo Lee, Chi Kyung Kim, Kyungmi Oh, Ho Geol Woo, Sung Hyuk Heo, Juneyoung Lee, Ji Sung Lee, Philip B Gorelick, Jae Guk Kim, Hee-Joon Bae

**Affiliations:** Department of Neurology, Soonchunhyang University Hospital Seoul, Soonchunhyang University College of Medicine, Seoul, Republic of Korea; Department of Translational Medicine, Graduate School of Medicine, Seoul National University College of Medicine, Seoul, Republic of Korea; Department of Neurology, Soonchunhyang University Hospital Seoul, Soonchunhyang University College of Medicine, Seoul, Republic of Korea; Department of Neurology, Soonchunhyang University Hospital Seoul, Soonchunhyang University College of Medicine, Seoul, Republic of Korea; Department of Neurology, Daejeon Eulji Medical Center, Eulji University School of medicine, Daejeon, Republic of Korea; Department of Neurology, Nowon Eulji Medical Center, Eulji University School of Medicine, Seoul, Republic of Korea; Department of Neurology, Dong-A University Hospital, Busan, Republic of Korea; Division of Intensive Care Medicine, Department of Neurosurgery and Neurology, Seoul National University Bundang Hospital, Seongnam, Republic of Korea; Department of Neurology, Seoul National University College of Medicine, Seoul National University Bundang Hospital, Seongnam, Republic of Korea; Department of Neurology, Seoul National University College of Medicine, Seoul National University Bundang Hospital, Seongnam, Republic of Korea; Department of Neurology, Seoul National University College of Medicine, Seoul National University Bundang Hospital, Seongnam, Republic of Korea; Department of Neurology, Seoul National University College of Medicine, Seoul National University Bundang Hospital, Seongnam, Republic of Korea; Department of Neurology, Seoul National University College of Medicine, Seoul National University Bundang Hospital, Seongnam, Republic of Korea; Department of Neurology, Seoul National University College of Medicine, Seoul National University Bundang Hospital, Seongnam, Republic of Korea; Department of Neurology, Seoul National University College of Medicine, Seoul National University Bundang Hospital, Seongnam, Republic of Korea; Department of Neurology, Seoul Medical Center, Seoul, Republic of Korea; Department of Neurology, Seoul Medical Center, Seoul, Republic of Korea; Department of Neurology, Yeungnam University Medical Center, Daegu, Republic of Korea; Department of Neurology, Yeungnam University Medical Center, Daegu, Republic of Korea; Department of Neurology, Inje University Ilsan Paik Hospital, Goyang, Republic of Korea; Department of Neurology, Inje University Ilsan Paik Hospital, Goyang, Republic of Korea; Department of Neurology, Inje University Ilsan Paik Hospital, Goyang, Republic of Korea; Department of Neurology, Hallym University Sacred Heart Hospital, Anyang, Republic of Korea; Department of Neurology, Hallym University Sacred Heart Hospital, Anyang, Republic of Korea; Department of Neurology, Hallym University Sacred Heart Hospital, Anyang, Republic of Korea; Department of Neurology, Hallym University Sacred Heart Hospital, Anyang, Republic of Korea; Department of Neurology, Hallym University Sacred Heart Hospital, Anyang, Republic of Korea; Department of Neurology, Chonnam National University Hospital, Gwangju, Republic of Korea; Department of Neurology, Chonnam National University Hospital, Gwangju, Republic of Korea; Department of Neurology, Chonnam National University Hospital, Gwangju, Republic of Korea; Department of Neurology, Dongguk University Ilsan Hospital, Goyang, Republic of Korea; Department of Neurology, Dongguk University Ilsan Hospital, Goyang, Republic of Korea; Department of Neurology, Jeju National University Hospital, Jeju, Republic of Korea; Department of Neurology, Jeju National University Hospital, Jeju, Republic of Korea; Departments of Neurology, Chungbuk National University Hospital, Chungbuk National University College of Medicine, Cheongju, Republic of Korea; Departments of Neurology, Chungbuk National University Hospital, Chungbuk National University College of Medicine, Cheongju, Republic of Korea; Department of Neurology, Ulsan University Hospital, Ulsan, Republic of Korea; Department of Neurology, Ulsan University Hospital, Ulsan, Republic of Korea; Department of Neurology, Keimyung University Dongsan hospital, Daegu, Republic of Korea; Department of Neurology, Keimyung University Dongsan hospital, Daegu, Republic of Korea; Department of Neurology, Keimyung University Dongsan hospital, Daegu, Republic of Korea; Department of Neurology, Hallym University Chuncheon Sacred Heart Hospital, Chuncheon, Republic of Korea; Department of Neurology, Hallym University Chuncheon Sacred Heart Hospital, Chuncheon, Republic of Korea; Department of Neurology, Chung-Ang University Hospital, Seoul, Republic of Korea; Department of Neurology, Chung-Ang University Hospital, Seoul, Republic of Korea; Department of Neurology, Chung-Ang University Hospital, Seoul, Republic of Korea; Department of Neurology, Uijeongbu Eulji Medical Center, Eulji University School of Medicine, Uijenongbu, Republic of Korea; Department of Neurology, Uijeongbu Eulji Medical Center, Eulji University School of Medicine, Uijenongbu, Republic of Korea; Department of Neurology, Uijeongbu Eulji Medical Center, Eulji University School of Medicine, Uijenongbu, Republic of Korea; Department of Radiology, Korea University Guro Hospital, Seoul, Republic of Korea; Department of Neurology, Korea University Guro Hospital, Seoul, Republic of Korea; Department of Neurology, Korea University Guro Hospital, Seoul, Republic of Korea; Department of Neurology, Korea University Guro Hospital, Seoul, Republic of Korea; Department of Neurology, Kyung Hee University Hospital, Seoul, Republic of Korea; Department of Neurology, Kyung Hee University Hospital, Seoul, Republic of Korea; Korea University, BK21FOUR R&E Center for Learning Health Systems, Department of Biostatistics, Seoul, Republic of Korea; Asan Medical Center, Clinical Research Center, Seoul, Republic of Korea; Davee Department of Neurology, Northwestern University Feinberg School of Medicine, Chicago, Illinois, United States; Department of Neurology, Daejeon Eulji Medical Center, Eulji University School of medicine, Daejeon, Republic of Korea; Department of Neurology, Seoul National University College of Medicine, Seoul National University Bundang Hospital, Seongnam, Republic of Korea

**Keywords:** basilar artery occlusion, endovascular treatment, real-world, stroke

## Abstract

**Background:**

The ATTENTION (0–12 hours) and BAOCHE (6–24 hours) trials demonstrated the efficacy of endovascular treatment (EVT) for basilar artery occlusion (BAO) in highly selected populations, but real-world generalizability remains uncertain.

**Patients and methods:**

We analyzed patients with acute BAO presenting within 24 hours of last known well from a nationwide, multicenter, prospective stroke registry. Patients were categorised as ATTENTION-eligible, BAOCHE-eligible, and ineligible based on clinical and neuroimaging criteria from both trials. The primary outcome was the distribution of 3-month modified Rankin Scale (mRS). Secondary outcomes included 3-month mRS of 0–3 and 0–2, 90-day mortality, and symptomatic hemorrhagic transformation. Inverse probability of treatment weighting was applied to adjust for baseline differences.

**Results:**

Among 49,471 patients with acute ischemic stroke, 2.0% (*n* = 1012) had BAO. Of these, 24% met ATTENTION-criteria, 6% met BAOCHE-criteria, and 72% were ineligible for both. Endovascular treatment was performed in 75%, 59%, and 43% of these groups, respectively. In ATTENTION-eligible patients, EVT was associated with more favourable mRS distribution (cOR, 1.73; 95% CI, 1.03–2.93) and lower 3-month mortality (RR, 0.52; 95% CI, 0.33–0.80). In BAOCHE-eligible patients, adjusted models did not reach statistical significance due to limited sample size. Among ineligible patients, EVT was associated with lower mortality (RR, 0.77; 95% CI, 0.60–0.99). Symptomatic haemorrhage rates did not differ across groups.

**Conclusion:**

Most BAO patients were ineligible for pivotal EVT trials, and eligibility for the more stringent BAOCHE criteria was particularly rare in real-world practice. Endovascular treatment was associated with reduced mortality even in ineligible patients. These findings highlight the practical limitations of current trial-based selection criteria and support broader application of EVT for BAO.

## Graphical Abstract

**Graphical Abstract:**
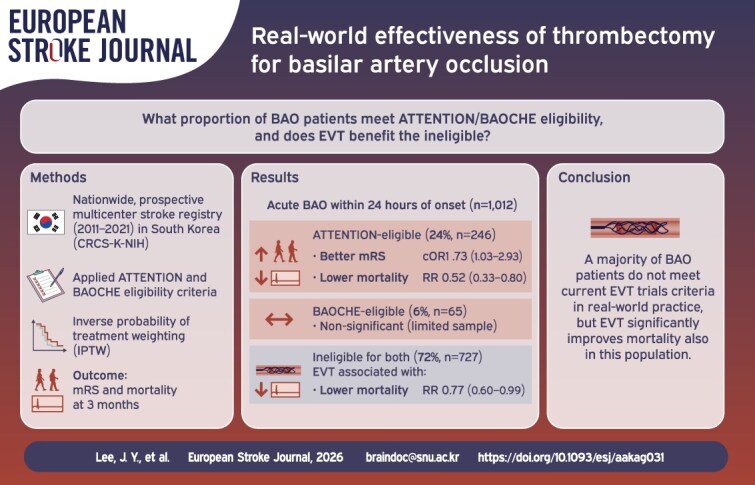

## Introduction

Two pivotal randomised trials, ATTENTION (0–12 hours)^1^ and BAOCHE (6–24 hours),^2^ demonstrated the efficacy of endovascular treatment (EVT) in selected patients with basilar artery occlusion (BAO). These positive findings contrasted with the negative results of earlier trials, BEST^3^ and BASICS.^4^ Meta-analyses combining these trials have confirmed the superiority of EVT over best medical management.^5–8^

Randomised trials are critical for establishing treatment efficacy, generally including highly selected populations treated at high-volume centres with specialised resources.^9^^,^^10^ Importantly, the strict eligibility criteria in these trials, such as minimum National Institutes of Health Stroke Scale (NIHSS) scores and neuroimaging criteria, are designed to optimise study validity, and their uncritical adoption as rigid clinical gatekeeping tools may unnecessarily exclude patients who could benefit from treatment in real-world practice.^11^ Indeed, Even before the ATTENTION and BAOCHE results—despite earlier negative trials—international stroke guidelines indicated that EVT may be considered for carefully selected BAO patients.^12^ Given the poor prognosis of BAO, clinicians continued to perform EVT on a case-by-case basis according to their experience and clinical judgement—often 24 hours or more after symptom onset—rather than strictly adhering to trial-based eligibility criteria.^13^^,^^14^

This gap between trial-based evidence^1^^,^^2^ and real-world clinical practice^15^^,^^16^ provides an opportunity to evaluate the differential effectiveness of EVT according to trial eligibility. Using a nationwide, prospective, multicentre stroke registry, we aimed to estimate the proportion of BAO patients who would have met the eligibility criteria of ATTENTION and BAOCHE and to assess the effectiveness of EVT across these groups.

## Methods

### Study design and population

We conducted a retrospective analysis of the Clinical Research Collaboration for Stroke in Korea—National Institute of Health (CRCS-K-NIH) registry, a nationwide, multicentre, stroke registry established in 2008. The registry prospectively enrols consecutive patients admitted with acute stroke or transient ischemic attack (TIA) on a voluntary basis. Detailed information about the CRCS-K-NIH registry has been published previously.^17^

The study included adult patients with acute ischemic stroke (AIS) and clinically relevant BAO, confirmed by angiography, who presented to the emergency department within 24 hours of their time last known well (TLKW) between January 2011 and December 2021. Patients were excluded if they (1) had been transferred after undergoing EVT at another hospital or (2) lacked neuroimaging data during hospitalisation or information on pre-stroke functional status.

To evaluate the effectiveness of EVT for BAO, patients were categorised into three groups based on the eligibility criteria used in the ATTENTION and BAOCHE trials (Table S1): ATTENTION-eligible, BAOCHE-eligible, and ineligible for both trials.

### Assessment of baseline neuroimaging parameters used in ATTENTION and BAOCHE trials

We collected all neuroimaging studies obtained within 24 hours of patient arrival. Baseline images were selected using the following criteria: (1) pre-treatment brain images in EVT-treated patients, or (2) brain images obtained within 24 hours of arrival in non-EVT patients. In accordance with the BAOCHE trial protocol,^2^ diffusion-weighted imaging (DWI) was preferred over computed tomography angiography (CTA) source images and non-contrast computed tomography (NCCT), and CTA source images were preferred over NCCT when DWI was unavailable. If multiple scans of the same modality were available, the earliest image was used as the baseline study.

For each patient, the Posterior Circulation Acute Stroke Prognosis Early CT Score (PC-ASPECTS; range, 0–10; lower scores indicate larger infarct volume)^18^ and pons-midbrain index (range, 0–8; higher scores indicate greater infarct burden)^19^ were assessed using the baseline images. Eighteen vascular neurologists or neuroradiologists evaluated images. To ensure consistency among raters, coordination meetings were held before and midway through the review process. Each image was independently reviewed by two raters blinded to clinical information. In case of disagreement, the final PC-ASPECTS and pons-midbrain index scores were adjudicated by an imaging core laboratory (K.J.G. and L.J.Y.). Interobserver agreement, assessed by a weighted Cohen’s Kappa, was 0.46 for PC-ASPECTS and 0.50 for the pons-midbrain index (Figure S1).

### Study outcomes

To evaluate the effectiveness of EVT for BAO, outcome measures included the proportions of patients with 3-month modified Rankin Scale (mRS) scores of 0–3 and 0–2; the shift in 3-month mRS; 3-month mortality; and the occurrence of symptomatic intracerebral haemorrhage (ICH) during hospitalisation.

In accordance with the registry protocol,^17^ trained research coordinators at each participating centre—certified via a standardised web-based education system (stroke-edu.or.kr)—prospectively collected 3-month outcome data following the index stroke. Outcomes were assessed using structured telephone interviews and medical record reviews. To ensure data quality, systematic audits, including regular on-site visits and source document verification, were conducted by an independent outcome adjudication committee.

### Statistical analysis

Categorical variables were compared using Pearson’s chi-square test or Fisher’s exact test, and continuous variables were analyzed using Student’s *t*-test or Wilcoxon rank-sum test, as appropriate.

Missing data for initial glucose levels (*n* = 7; 0.69%) were imputed with group median values. For missing 3-month mRS scores (*n* = 44, 4.35%), values were imputed using the 1-year mRS score (*n* = 10) or the discharge mRS score (*n* = 34).

The effectiveness of EVT was estimated using modified Poisson regression for binary outcomes and ordinal logistic regression for the mRS distribution, incorporating generalised estimating equations to account for clustering by centre. Covariates were pre-specified for each model.^15^^,^^20^^,^^21^ For ATTENTION-eligible and trial-ineligible groups, adjustments were made for the following variables: age, sex, diabetes, history of stroke or TIA, initial NIHSS, pre-stroke mRS, causative mechanism (TOAST, Trial of ORG 10172 in Acute Stroke Treatment classification),^22^ intravenous thrombolysis (IVT) administration, systolic blood pressure, transfer-in status, time from TLKW to arrival, and PC-ASPECTS. In the BAOCHE-eligible group, only age was adjusted for due to the limited sample size.

To further address baseline imbalances between EVT-treated and non-treated patients, stabilised inverse probability of treatment weighting (IPTW) analysis was applied using propensity scores derived from clinically relevant variables. These included all variables from the multivariable models as well as hypertension, dyslipidemia, smoking status, coronary artery disease, atrial fibrillation, pons-midbrain index, and initial serum glucose.^15^^,^^21^

Two sensitivity analyses were performed. First, the primary analyses were repeated after excluding patients with missing 3-month outcome data. Second, in the IPTW analysis, an interaction term between EVT and admission epoch was introduced to assess effect modification over time. Admission epochs were categorised as: 2011-2014, 2015-2018, and 2019-2021.

A two-sided *P*-value of <.05 was considered statistically significant. All statistical analyses were performed using SAS version 9.4 (SAS Institute, Cary, NC, United States) and R software version 4.3.2 (R Foundation for Statistical Computing, Vienna, Austria).

Results

### Study population

**Figure 1:**
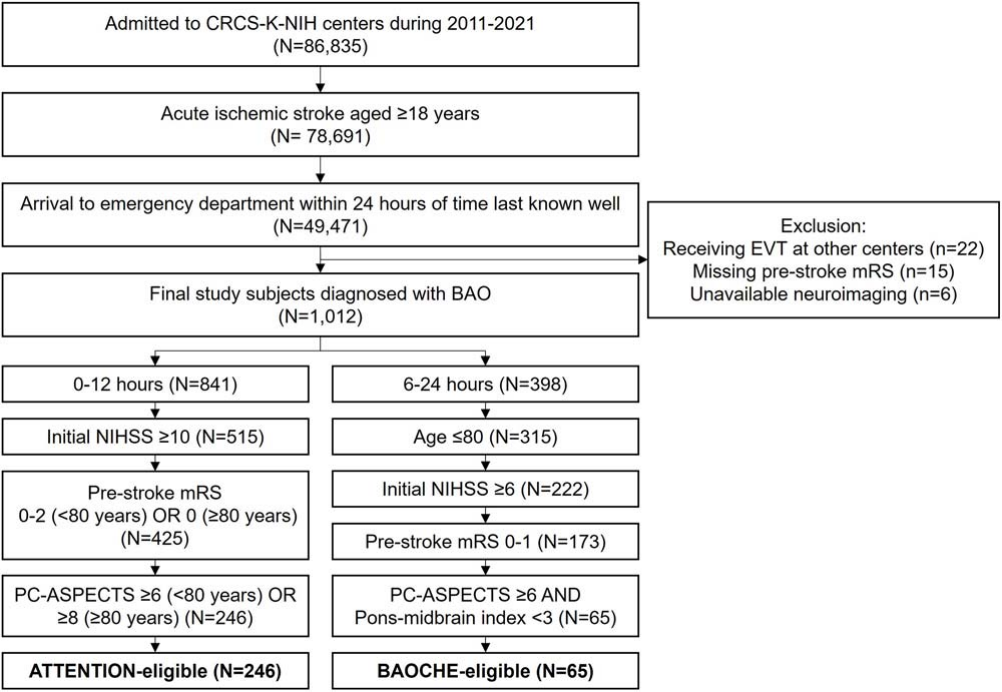
Flow diagram of patient inclusion and trial eligibility classification. Abbreviations: BAO, basilar artery occlusion; mRS, modified Rankin Scale; NIHSS, National Institutes of Health Stroke Scale; PC-ASPECTS, Posterior Circulation Acute Stroke Prognosis Early CT Score.

Among 49,471 adult patients who presented to the emergency department within 24 hours of TLKW, 2% (*n* = 1,012) were diagnosed with AIS and clinically relevant BAO (Figure 1). Initial cohorts of 841 (ATTENTION time window) and 398 (BAOCHE time window) patients were reduced to 246 and 65 eligible patients, respectively. Beyond presentation time and age cutoffs, the two most frequent reasons for trial exclusion were failing to meet baseline NIHSS cutoffs (<10 and <6, respectively) and prespecified neuroimaging thresholds.

The mean age was 70.8 ± 12.0 years, with 59.8% (*n* = 605) being male. The median initial NIHSS was 13 (IQR 6–22), and 51.3% (*n* = 519) underwent EVT. The median time from TLKW to hospital arrival was 4 hours. On neuroimaging, the median PC-ASPECTS was 7 (IQR 5–8), and the median pons-midbrain index was 2 (IQR 0–3). (Table 1).

**Table 1:** Baseline characteristics of patients with basilar artery occlusion, stratified by trial eligibility.

	All patients	Eligible for ATTENTION	Eligible for BAOCHE	Ineligible for both trials
***N***	1012	246	65	727
**Demographic factor**				
** age, mean, SD, y**	70.8 ± 12.0	69.0 ± 11.3	67.2 ± 10.8	71.6 ± 12.2
** male, *n* (%)**	605 (59.8)	158 (64.2)	46 (70.8)	419 (57.6)
**History of medical illness**				
** Hypertension, *n* (%)**	709 (70.1)	170 (69.1)	48 (73.9)	510 (70.2)
** Diabetes, *n* (%)**	296 (29.3)	70 (28.5)	24 (36.9)	211 (29.0)
** Dyslipidemia, *n* (%)**	288 (28.5)	73 (29.7)	20 (30.8)	205 (28.2)
** Smoking, *n* (%)**	338 (33.4)	94 (38.2)	32 (49.2)	226 (31.1)
** Stroke or TIA, *n* (%)**	244 (24.1)	42 (17.1)	12 (18.5)	195 (26.8)
** CAD, *n* (%)**	113 (11.2)	27 (11.0)	4 (6.2)	84 (11.6)
** Atrial fibrillation, *n* (%)**	394 (38.9)	101 (41.1)	19 (29.2)	280 (38.5)
**Initial NIHSS, median (IQR)**	13 (6–22)	19 (14–24)	11 (7–19)	9 (4–21)
** 0–5, *n* (%)**	251 (24.8)	0 (0)	0 (0)	251 (34.5)
**Pre-stroke mRS**				
** 0, *n* (%)**	763 (75.4)	222 (90.2)	62 (95.4)	503 (69.2)
** 1, *n* (%)**	77 (7.6)	17 (6.9)	3 (4.6)	59 (8.1)
** ≥2, *n* (%)**	172 (17)	7 (2.8)	0 (0)	165 (22.7)
**Causative mechanism**				
** Large artery atherosclerosis, *n* (%)**	354 (35.0)	79 (32.1)	34 (52.3)	256 (35.2)
** Cardioembolism, *n* (%)**	390 (38.5)	105 (42.7)	17 (26.2)	274 (37.7)
** Other determined, *n* (%)**	14 (1.4)	3 (1.2)	2 (3.1)	10 (1.4)
** Undetermined, *n* (%)**	254 (25.1)	59 (24.0)	12 (18.5)	187 (25.7)
**PC-ASPECTS, median (IQR)**	7 (5–8)	8 (7–9)	7(6–9)	6 (4–8)
** <6, *n* (%)**	288 (28.5)	0 (0)	0 (0)	288 (39.6)
**Pons-midbrain index, median (IQR)**	2 (0–3)	0 (0–2)	1 (0–2)	2 (0–4)
** ≥3, *n* (%)**	319 (31.5)	49 (19.9)	0 (0)	270 (37.1)
**SBP, median (IQR)**	148 (130–165)	147 (130–164)	148 (130–162)	148 (130–165)
**Initial glucose, median (IQR)**	120 (119–159)	119 (119–161)	119 (119–161)	120 (119–159)
**IVT, *n* (%)**	320 (31.6)	137 (55.7)	7 (10.8)	179 (24.6)
**EVT, *n* (%)**	519 (51.3)	185 (75.2)	38 (58.5)	314 (43.2)
**Transfer-in, *n* (%)**	47 (4.6)	12 (4.9)	1 (1.5)	34 (4.7)
**Workflow time metrics**				
** TLKW to arrival, median (IQR), hours**	4 (2–9)	2 (1–5)	9 (7–13)	5 (2–11)
** Arrival to puncture, median (IQR), mins**	109 (82–148)	102 (73–130)	108 (79–131)	114 (86–183)

Values are presented as mean ± SD, median (IQR), or *n* (%), as appropriate.

Abbreviations: CAD, coronary artery disease; EVT, endovascular thrombectomy; IQR, interquartile range; IVT, intravenous thrombolysis; mRS, modified Rankin Scale; NIHSS, National Institutes of Health Stroke Scale; PC-ASPECTS, Posterior Circulation Acute Stroke Prognosis Early CT Score; SBP, systolic blood pressure; TIA, transient ischemic attack; TLKW, time last known well.

Percentages may not total 100% due to rounding.

### Trial eligibility and EVT rates

Of the 1,012 AIS patients with BAO, 24.3% (*n* = 246) met the ATTENTION trial criteria, 6.4% (*n* = 65) met the BAOCHE criteria, and 26 patients met both criteria (Figure 1). The remaining 72.0% (*n* = 727) were ineligible for both trials.

Compared with trial-eligible patients, ineligible patients were older, more often women and non-smokers, had a higher frequency of recurrent stroke, and exhibited milder stroke severity, greater pre-stroke disability, and more unfavourable neuroimaging parameters (Table S2). Notably, BAOCHE-eligible patients were younger, had a higher prevalence of large artery atherosclerosis, and presented later than both ATTENTION-eligible and trial-ineligible patients (Table 1).

**Figure 2:**
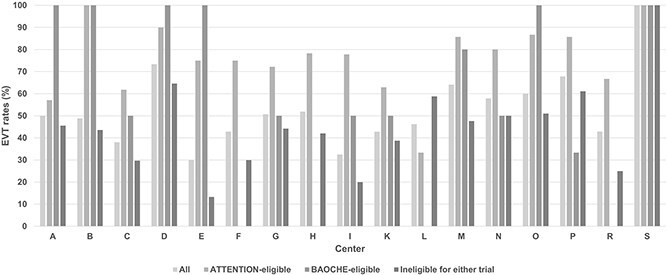
Endovascular treatment rates by center and trial eligibility. Each bar represents the percentage of patients undergoing EVT at each center, stratified by ATTENTION-eligible, BAOCHE-eligible, and ineligible groups.

Endovascular treatment was performed in 75.2% of ATTENTION-eligible, 58.5% of BAOCHE-eligible, and 43.2% of trial-ineligible patients. EVT rates varied substantially across centres (Figure 2 and Table S3), ranging from 33.3% to 100% in ATTENTION-eligible patients, from 0% to 100% in BAOCHE-eligible patients, and from 13.3% to 100% among trial-ineligible patients.

### Effectiveness of EVT in ATTENTION-eligible patients

Among ATTENTION-eligible patients, age and time from TLKW to hospital arrival were similar between EVT and non-EVT groups. Patients treated with EVT were more likely to be men, have atrial fibrillation, lower pre-stroke disability, more severe stroke, more favourable neuroimaging parameters, and higher IVT rates (Table 2).

**Table 2:** Baseline characteristics of EVT-treated and non-treated patients, stratified by trial eligibility.

	ATTENTION-eligible (*n* = 246)			BAOCHE-eligible (*n* = 65)			Ineligible for both trials (*n* = 727)		
	EVT	No EVT	*P* ^a^	EVT	No EVT	*P* ^a^	EVT	No EVT	*P* ^a^
***N***	185	61		38	27		314	413	
**Demographic factor**									
** age, mean, SD, y**	68.8 ± 11.6	69.3 ± 10.4	.76	65.5 ± 11.0	69.5 ± 10.3	.15	70.8 ± 13.1	72.2 ± 11.6	.14
** male, *n* (%)**	122 (65.9)	36 (59.0)	.33	28 (73.7)	18 (66.7)	.54	186 (59.2)	233 (56.4)	.45
**History of medical illness**									
** Hypertension, *n* (%)**	125 (67.6)	45 (73.8)	.36	26 (68.4)	22 (81.5)	.24	212 (67.5)	298 (72.2)	.18
** Diabetes, *n* (%)**	48 (25.9)	22 (36.1)	.13	12 (31.6)	12 (44.4)	.29	90 (28.7)	121 (29.3)	.85
** Dyslipidemia, *n* (%)**	55 (29.7)	18 (29.5)	.97	13 (34.2)	7 (25.9)	.48	84 (26.8)	121 (29.3)	.45
** Smoking, *n* (%)**	70 (37.8)	24 (39.3)	.83	20 (52.6)	12 (44.4)	.52	90 (28.7)	136 (32.9)	.22
** Stroke or TIA, *n* (%)**	28 (15.1)	14 (23.0)	.16	6 (15.8)	6 (22.2)	.53	69 (22.0)	126 (30.5)	.01
** CAD, *n* (%)**	25 (13.5)	2 (3.3)	.03	2 (5.3)	2 (7.4)	>.99	31 (9.9)	53 (12.8)	.22
** Atrial fibrillation, *n* (%)**	80 (43.2)	21 (34.4)	.23	11 (28.9)	8 (29.6)	.95	138 (43.9)	142 (34.4)	.01
**Initial NIHSS, median (IQR)**	19 (14–25)	17 (13–21)	.02	12 (7–19)	9 (7–18)	.15	14 (6–23)	7 (3–19)	<.001
**Pre-stroke mRS**			.15			>.99			.13
** 0, *n* (%)**	169 (91.4)	53 (86.9)		36 (94.7)	26 (96.3)		228 (72.6)	275 (66.6)	
** 1, *n* (%)**	13 (7.0)	4 (6.6)		2 (5.3)	1 (3.7)		26 (8.3)	33 (8.0)	
** ≥2, *n* (%)**	3 (1.6)	4 (6.6)		0 (0.0)	0 (0.0)		60 (19.1)	105 (25.4)	
**Causative mechanism**			.33			.85			.02
**Large artery atherosclerosis, *n* (%)**	54 (29.2)	25 (41.0)		20 (52.6)	14 (51.9)		93 (29.6)	163 (39.5)	
** Cardioembolism, *n* (%)**	83 (44.9)	22 (36.1)		11 (28.9)	6 (22.2)		137 (43.6)	137 (33.2)	
** Other determined, *n* (%)**	3 (1.6)	0 (0.0)		1 (2.6)	1 (3.7)		4 (1.3)	6 (1.5)	
** Undetermined, *n* (%)**	45 (24.3)	14 (23.0)		6 (15.8)	6 (22.2)		80 (25.5)	107 (25.9)	
**PC-ASPECTS, median (IQR)**	8 (7–10)	8 (7–9)	.03	8 (7–9)	7 (6–8)	.19	6 (4–8)	7 (5–8)	<.001
**Pons-midbrain index,** **median (IQR)**	0 (0–2)	1 (0–2)	.07	0 (0–1)	1 (0–2)	.08	2 (1–4)	2 (0–3)	<.001
**SBP (IQR)**	147 (130–164)	140 (130–161)	.70	148 (130–160)	149 (130–170)	.72	148 (130–164)	150 (130–166)	.86
**Initial glucose, median (IQR)**	119 (119–153)	120 (119–161)	.09	119 (119–161)	120 (119–163)	.12	120 (119–159)	120 (119–159)	.31
**IVT, *n* (%)**	106 (57.3)	31 (50.8)	.38	4 (10.5)	3 (11.1)	>.99	92 (29.3)	87 (21.1)	.01
**mTICI 2b-3, *n* (%)**	152 (82.2)	NA		31 (81.6)	NA		235 (74.8)	NA	
**Transfer-in, *n* (%)**	10 (5.4)	2 (3.3)	.74	0 (0.0)	1 (3.7)	.42	22 (7.0)	12 (2.9)	.01
**Workflow time metrics**									
**TLKW to arrival, median (IQR), hours**	2 (1–5)	3 (1-5)	.40	9 (7–13)	10 (7-13)	.97	4 (2–9)	6 (2–12)	<.001
**Arrival to EVT, median (IQR), mins**	102 (73–130)	-		108 (79–132)	-		114 (86–183)	-	

Values are presented as mean ± SD, median (IQR), or *n* (%), as appropriate.

mTICI, modified Thrombolysis in Cerebral Infarction. See Table 1 footnote for abbreviation definitions.

a *P* values were calculated using Student *t* test, Wilcoxon rank-sum test, χ^2^ test, or Fisher exact test, as appropriate.

**Figure 3:**
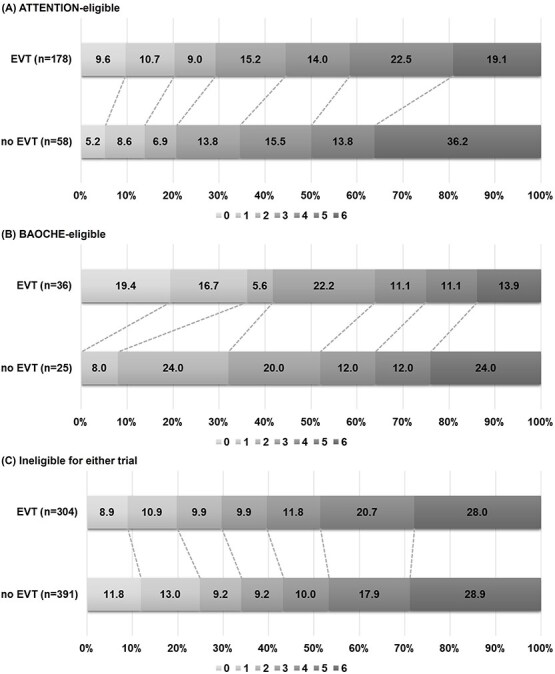
Distribution of 3-month mRS by EVT and eligibility group. Distribution of 3-month mRS scores in EVT-treated vs non-treated patients, stratified by trial eligibility. Values shown are unadjusted percentages without missing imputation. Abbreviations: EVT, endovascular treatment; mRS, modified Rankin Scale.

A trend towards improved 3-month mRS distribution was observed in the EVT group (*P* = .06, Table 3 and Figure 3), along with a lower 3-month mortality rate. Symptomatic ICH rates were similar between groups. After applying stabilised IPTW (Figure S2), EVT was associated with higher odds of favourable mRS (cOR, 1.73; 95% CI, 1.03–2.93) and lower 3-month mortality (RR, 0.52; 95% CI, 0.33–0.80).

**Table 3:** Clinical outcomes according to EVT status and trial eligibility.

	ATTENTION-eligible (N = 246)				
	EVT (*n* = 185)	No EVT (*n* = 61)	*P* ^a^	Before IPTW	After IPTW
				RR/cOR (95% CI)^b^	RR/cOR (95% CI)^c^
**mRS at 3 months**					
**mRS 0–3, *n* (%)**	81 (43.8)	22 (36.1)	.29	1.16 (0.80–1.69)	1.25 (0.84–1.87)
**mRS 0–2, *n* (%)**	53 (28.6)	14 (23.0)	.39	1.19 (0.70–2.01)	1.16 (0.68–1.99)
**ordinal mRS, median (IQR)**	4 (2–5)	4 (3–6)	.09	1.45 (0.83–2.53)	1.73 (1.03–2.93)
**Mortality at 3 months, *n* (%)**	34 (18.4)	21 (34.4)	.01	0.58 (0.36–0.93)	0.52 (0.33–0.80)
**Symptomatic ICH, *n* (%)**	7 (3.8)	3 (4.9)	.71	NA	NA
	**BAOCHE-eligible (*n* = 65)**				
	**EVT** **(*n* = 38)**	**No EVT** **(*n* = 27)**	***P* ^a^**	**Before IPTW**	**After IPTW**
				**RR/cOR** **(95% CI)^d^**	**RR/cOR** **(95% CI)**
**mRS at 3 months**					
**mRS 0–3, *n* (%)**	23 (60.5)	15 (55.6)	.69	1.00 (0.67–1.48)	NA
**mRS 0–2, *n* (%)**	15 (39.5)	10 (37.0)	.84	1.01 (0.53–1.90)	NA
**ordinal mRS, median (IQR)**	3 (1–5)	3 (2–5)	.30	1.47 (0.61–3.56)	NA
**Mortality at 3 months, *n* (%)**	5 (13.2)	6 (22.2)	.50	0.65 (0.24–1.73)	NA
**Symptomatic ICH, *n* (%)**	1 (2.6)	0 (0.0)	1.00	NA	NA
	**Ineligible for both trials (*n* = 727)**				
	**EVT** **(*n* = 314)**	**No EVT** **(*n* = 413)**	***P* ^a^**	**Before IPTW**	**After IPTW**
				**RR/cOR** **(95% CI)^b^**	**RR/cOR** **(95% CI)^c^**
**mRS at 3 months**					
**mRS 0–3, *n* (%)**	121 (38.5)	171 (41.4)	.43	1.27 (1.09–1.47)	1.13 (0.94–1.37)
**mRS 0–2, *n* (%)**	91 (29.0)	135 (32.7)	.28	1.16 (0.96–1.40)	1.03 (0.81–1.30)
**ordinal mRS, median (IQR)**	4 (2–6)	4 (2–6)	.55	1.46 (1.10–1.96)	1.22 (0.94–1.59)
**Mortality at 3 months, *n* (%)**	85 (27.1)	116 (28.1)	.76	0.86 (0.68–1.09)	0.77 (0.60–0.99)
**Symptomatic ICH, *n* (%)**	6 (1.9)	9 (2.2)	.80	NA	0.96 (0.33–2.81)

Abbreviations: cOR, common odds ratio; CI, confidence interval; ICH, intracranial haemorrhage; IQR, interquartile range; IPTW, inverse probability of treatment weighting; mRS, modified Rankin Scale; NA, not applicable; RR, risk ratio.

a *P*-value by Chi-square test, Fisher’s exact test, and Cochran–Mantel–Haenszel shift test

b Modified Poisson regression or ordinal logistic regression for lower mRS was applied adjusting for age, sex, diabetes, history of stroke or transient ischemic attack, initial stroke severity, pre stroke mRS, causative mechanism, intravenous thrombolysis, systolic blood pressure, transfer-in status, elapsed time from time last known well to arrival, and PC-ASPECTS.

c Weighted modified Poisson regression or ordinal logistic regression with robust standard errors was applied, adjusting for variables showing imbalances after applying stabilised IPTW.

d Modified Poisson regression or ordinal logistic regression for lower mRS was applied adjusting for age.

### Effectiveness of EVT in BAOCHE-eligible patients

Among BAOCHE-eligible patients, baseline characteristics were balanced between EVT and non-EVT groups (Table 2). A non-significant trend towards improved 3-month mRS distribution was observed with EVT (*P* = .10, Table 3 and Figure 3), along with numerically lower 3-month mortality (13.9% vs 24.0%, *P* = .33).

Adjusted models did not reach statistical significance, including 3-month mRS distribution (cOR, 1.47; 95% CI, 0.61–3.56) and mortality (RR, 0.65; 95% CI, 0.24–1.73).

### Effectiveness of EVT in patients ineligible for both trials

Among trial-ineligible patients, age, sex distribution, and pre-stroke mRS were comparable between EVT and non-EVT groups. EVT recipients were more likely to have atrial fibrillation, more severe stroke, more favourable neuroimaging features, earlier presentation, higher IVT rates, and be transferred in from another hospital (Table 2).

In unadjusted analyses, there were no significant differences in functional outcomes or mortality (Table 3 and Figure 3). However, in adjusted models, EVT was associated with higher likelihood of achieving 3-month mRS 0-3 and a more favourable 3-month mRS distribution.

After applying stabilised IPTW, covariate balance between EVT and non-EVT groups was achieved (Figure S3). In this model, EVT was significantly associated with lower 3-month mortality (cOR, 0.77; 95% CI, 0.60-0.99). Although associations with functional outcomes did not reach statistical significance, the point estimates were numerically greater than unity for both 3-month mRS 0–3 (RR, 1.13; 95% CI, 0.94–1.37) and the ordinal 3-month mRS distribution (cOR, 1.22; 95% CI, 0.94–1.59).

### Sensitivity analyses

After excluding 44 patients with missing 3-month mRS data, sensitivity analyses yielded consistent results for ATTENTION-eligible and BAOCHE-eligible patients (Table S4). In IPTW-adjusted analyses of trial-ineligible patients (*n* = 695), EVT was associated with higher likelihood of achieving 3-month mRS 0-3 (RR, 1.25; 95% CI, 1.07–1.47) and a favourable 3-month mRS distribution (cOR, 1.40; 95% CI, 1.06–1.86). A consistent reduction in 3-month mortality by EVT was also observed (RR, 0.79; 95% CI, 0.63–1.00; *P* = .048).

### Effect modification of EVT by time period

In both ATTENTION-eligible and trials-ineligible groups, no significant effect modification of EVT was observed across admission epochs (2011-2014, 2015-2018, and 2019-2021) (Table S5).

## Discussion

Our finding that BAO accounted for 2% of AIS cases presenting within 24 hours of TLKW is consistent with prior literature.^5^ Among these patients, more than 70% did not meet the eligibility criteria for either of the two pivotal EVT trials. Only 24.3% and 6.4% met the eligibility criteria for ATTENTION and BAOCHE, respectively. Among ATTENTION-eligible patients, EVT was associated with a statistically significant improvement in 3-month mRS distribution and a reduction in 3-month mortality.

Among BAOCHE-eligible patients, a non-significant trend towards improved 3-month mRS distribution was observed, but did not reach statistical significance in the adjusted models, likely due to limited sample size. This limited sample size (6.4% among BAO patients) in our real-world cohort reflects the reality that BAO patients meeting the major BAOCHE eligibility criteria—presentation within 6–24 hours, initial NIHSS ≥6, PC-ASPECTS ≥6, and pons–midbrain index <3—are not frequently encountered in clinical practice.

Notably, even among patients ineligible for both trials, EVT was associated with a significant mortality reduction, supporting its applicability beyond strict trial-based selection. While the association with functional outcome (mRS 0-3) did not reach statistical significance in this group after IPTW adjustment, the marked reduction in mortality (cOR, 0.77) is clinically profound. These findings suggest that EVT may help shift the outcome distribution by reducing the proportion of patients who would otherwise face certain death, potentially moving them into states of survived disability. Given that many patients and caregivers perceive severe disability (mRS 5) as a condition equivalent to or even worse than death,^23^ the survival benefit observed here warrants a nuanced discussion on the goals of care in trial-ineligible BAO patients.

Following the introduction of breakthrough therapies in acute stroke, such as IVT, it may be expected for exclusion criteria to become less stringent over time, as initial thresholds may prove overly restrictive in real-world practice.^24^ EVT consistently yielded favourable outcomes among ATTENTION-eligible patients in our cohort, despite notable differences from the original trial population.^1^ The high rate of EVT (43%) among trial-ineligible patients in our registry highlights the existing clinical equipoise and the reality that clinicians are already looking beyond restrictive trial criteria. This “real-world expansion” suggests that rigid adherence to trial-based selection may unnecessarily deny potentially life-saving treatment to a large majority of BAO patients.^9^ As indications for EVT have expanded in anterior circulation stroke—including extended time windows^25–27^ and large infarcts^28^^,^^29^—it is reasonable and necessary to critically reassess the current eligibility thresholds for BAO, including age, pre-stroke disability, time window, and neuroimaging parameters.

Our study also highlights significant challenges in patient selection based on neuroimaging. The interobserver agreement for PC-ASPECTS (κ = 0.46) and the pons-midbrain index (κ = 0.50) was only moderate, even among experienced raters. This inherent subjectivity in human interpretation may lead to inconsistent trial eligibility and treatment decisions.^30^ Furthermore, recent studies suggest that EVT may benefit patients with low PC-ASPECTS,^31^ mild stroke severity (NIHSS < 10),^32^ or those who do not meet strict eligibility criteria.^33^^,^^34^ To minimise unnecessary exclusions and standardise BAO management, our results strongly advocate for the development and integration of more objective selection tools, such as AI-based automated scoring systems^35^ or accessible imaging modalities.^20^

This study has several limitations. First, interobserver agreement for neuroimaging assessment was only moderate, despite structured adjudication sessions. Second, we could not fully account for all anatomical and laboratory exclusion criteria used in randomised trials.^1^^,^^2^ However, our goal was to reflect real-world practice, where selection is often broader. Third, although no effect modification by time period was observed, our study spanned a decade (2011-2021), during which EVT techniques evolved. Furthermore, as the data collection ended in 2021, our findings may not fully capture the most contemporary shifts in clinical practice and device technologies over the past four years. Fourth, results in the BAOCHE-eligible patients were inconclusive due to limited sample size. Lastly, as with all retrospective studies, unmeasured confounding is possible. We were unable to fully capture clinicians’ rationale behind treatment decisions, particularly for withholding EVT.

In conclusion, in this nationwide, real-world cohort of BAO patients presenting within 24 hours of TLKW, more than 70% did not meet the eligibility criteria of pivotal EVT trials. Particularly, the notably low proportion of patients meeting the BAOCHE criteria (6%) underscores the practical limitations of these strict criteria. Although the full magnitude of treatment effects seen in the trials was not replicated, EVT was associated with significant benefits in ATTENTION-eligible patients and a mortality reduction with potential functional improvement in trial-ineligible patients. These findings support reevaluating and potentially broadening EVT eligibility criteria for BAO. Future efforts should focus on refining selection tools—such as advanced imaging modalities or artificial intelligence-based algorithms—to enhance diagnostic consistency and reflecting the diversity of real-world patients in future clinical trial designs.

## Supplementary Material

supplemental_material_NIBAO_20260120_ESJ_aakag031

## Data Availability

The data supporting the findings of this study are available from the corresponding author upon reasonable request.
