# Supplementary material for: Real-world effectiveness of thrombectomy for basilar artery occlusion: lessons beyond the ATTENTION and BAOCHE trials
Source: Eur Stroke J. 2026 May 13;11(5):aakag031. doi: 10.1093/esj/aakag031 (PMC13171037; doi:10.1093/esj/aakag031)
Supplement: supplemental_material_NIBAO_20260120_ESJ_aakag031 [file supplemental_material_nibao_20260120_esj_aakag031.docx]

**Supplementary Materials**

**Table S1. Eligibility criteria adapted from ATTENTION and BAOCHE trials**

**Table S2. Baseline characteristics by eligibility for randomized trials (N=1,012)**

**Table S3. Number and distribution of enrolled patients by trial eligibility across centers**

**Table S4. Comparison of outcomes and association with EVT by trial eligibility after excluding patients with missing 3-month mRS data**

**Table S5. Association of EVT with clinical outcomes by trial eligibility and epoch**

**Figure S1. Interobserver agreement for neuroimaging measures**

**Figure S2. Baseline characteristics before and after applying IPTW in patients eligible for ATTENTION trial**

**Figure S3. Baseline characteristics before and after applying IPTW in patients ineligible for both trials**

**Table S1. Eligibility criteria adapted from ATTENTION and BAOCHE trials**

|  | **ATTENTION** | **BAOCHE** |
| --- | --- | --- |
| Inclusion | 1) Time from stroke onset/last known well to randomization within 12 hours  2) Age ≥18  3) Initial NIHSS ≥10  4) Basilar artery occlusion confirmed by angiography  5) Pre stroke mRS 0-2 (<80 yrs) or 0 (≥80 yrs) | 1) Time from stroke onset/last known well to randomization within 6-24 hours  2) Age ≥18 and ≤ 80  3) Initial NIHSS ≥6  4) Basilar artery occlusion confirmed by angiography  5) Pre stroke mRS 0-1 |
| Exclusion | PC-ASPECTS <6 (<80 yrs) or <8 (≥80 yrs) | PC-ASPECTS <6  Pons-midbrain-index ≥3 |

Abbreviations: mRS, modified Rankin scale; NIHSS, national institutes of health stroke scale; PC-ASPECTS, posterior circulation acute stroke prognosis early CT score.

**Table S2. Baseline characteristics by eligibility for randomized trials (N=1,012)**

|  | **Eligible for either trial** | **Ineligible for both trials** | ***P**** |
| --- | --- | --- | --- |
| N | 285 | 727 |  |
| Demographic factor |  |  |  |
| age, mean, SD, y | 68.8±11.2 | 71.6±12.2 | .001 |
| male, n (%) | 186 (65.3) | 419 (57.6) | .03 |
| History of medical illness |  |  |  |
| Hypertension, n (%) | 199 (69.8) | 510 (70.2) | .92 |
| Diabetes, n (%) | 85 (29.8) | 211 (29.0) | .80 |
| Dyslipidemia, n (%) | 83 (29.1) | 205 (28.2) | .77 |
| Smoking, n (%) | 112 (39.3) | 226 (31.1) | .01 |
| Stroke or TIA, n (%) | 49 (17.2) | 195 (26.8) | .001 |
| Coronary disease, n (%) | 29 (10.2) | 84 (11.6) | .53 |
| Atrial fibrillation, n (%) | 114 (40.0) | 280 (38.5) | .66 |
| Initial NIHSS, median (IQR) | 17 (12 - 24) | 9 (4 - 21) | <.001 |
| 0-5, n (%) | 0 (0.0) | 251 (34.5) |  |
| Pre stroke mRS |  |  | <.001 |
| 0, n (%) | 260 (91.2) | 503 (69.2) |  |
| 1, n (%) | 18 (6.3) | 59 (8.1) |  |
| ≥2, n (%) | 7 (2.5) | 165 (22.7) |  |
| Causative mechanism |  |  | .82 |
| Large artery atherosclerosis, n (%) | 98 (34.4) | 256 (35.2) |  |
| Cardioembolism, n (%) | 116 (40.7) | 274 (37.7) |  |
| Other determined, n (%) | 4 (1.4) | 10 (1.4) |  |
| Undetermined, n (%) | 67 (23.5) | 187 (25.7) |  |
| PC-ASPECTS, median (IQR) | 8 (7 - 9) | 6 (4 - 8) | <.001 |
| Pons-midbrain index, median (IQR) | 0 (0 - 2) | 2 (0 - 4) | <.001 |
| Systolic blood pressure, median (IQR) | 147 (130-164) | 148 (130-165) | .76 |
| Initial glucose, median (IQR) | 119 (119-161) | 120 (119-159) | .86 |
| Intravenous thrombolysis, n (%) | 141 (49.5) | 179 (24.6) | <.001 |
| Endovascular treatment, n (%) | 205 (71.9) | 314 (43.2) | <.001 |
| Transfer-in, n (%) | 13 (4.6) | 34 (4.7) | .94 |
| Workflow time metrics |  |  |  |
| TLKW to arrival, median (IQR), hours | 3 (1-7) | 5 (2-11) | <.001 |
| Arrival to puncture, median (IQR), mins | 104 (74-131) | 114 (86-183) | <.001 |

Abbreviations: IQR, interquartile range; mRS, modified Rankin Scale; NIHSS, national institutes of health stroke scale; PC-ASPECTS, posterior circulation acute stroke prognosis early CT score; TIA, transient ischemic attack; TLKW, time last known well.

******P*-value by Student's t-test, Wilcoxon rank sum test, Chi-square test, and Fisher's exact test.

**Table S3. Number and distribution of enrolled patients by trial eligibility across centers**

| **Center** | **All patients** | **ATTENTION-eligible** | **BAOCHE-eligible** | **Ineligible for both trials** |
| --- | --- | --- | --- | --- |
| **A** | 18 (1.8) | 7 (2.8) | 1 (1.5) | 11 (1.5) |
| **B** | 43 (4.2) | 2 (0.8) | 2 (3.1) | 39 (5.4) |
| **C** | 142 (14.0) | 34 (13.8) | 12 (18.5) | 101 (13.9) |
| **D** | 124 (12.3) | 40 (16.3) | 9 (13.8) | 82 (11.3) |
| **E** | 20 (2.0) | 4 (1.6) | 1 (1.5) | 15 (2.1) |
| **F** | 14 (1.4) | 4 (1.6) | 1 (1.5) | 10 (1.4) |
| **G** | 71 (7.0) | 18 (7.3) | 2 (3.1) | 52 (7.2) |
| **H** | 77 (7.6) | 23 (9.3) | 6 (9.2) | 50 (6.9) |
| **I** | 40 (4.0) | 9 (3.7) | 2 (3.1) | 30 (4.1) |
| **K** | 222 (21.9) | 35 (14.2) | 10 (15.4) | 181 (24.9) |
| **L** | 26 (2.6) | 6 (2.4) | 4 (6.2) | 17 (2.3) |
| **M** | 39 (3.9) | 14 (5.7) | 5 (7.7) | 21 (2.9) |
| **N** | 38 (3.8) | 10 (4.1) | 2 (3.1) | 26 (3.6) |
| **O** | 65 (6.4) | 15 (6.1) | 2 (3.1) | 49 (6.7) |
| **P** | 59 (5.8) | 21 (8.5) | 3 (4.6) | 36 (5.0) |
| **R** | 7 (0.7) | 3 (1.2) | 0 (0) | 4 (0.6) |
| **S** | 7 (0.7) | 1 (0.4) | 3 (4.6) | 3 (0.4) |
| **Total** | 1,012 (100) | 246 (100) | 65 (100) | 727 (100) |

Indicated as n (%).

**Table S4. Comparison of outcomes and association with EVT by trial eligibility after excluding patients with missing 3-month mRS data**

|  | **ATTENTION-eligible (N=236)** | | | | |
| --- | --- | --- | --- | --- | --- |
|  | **EVT**  **(N=178)** | **No EVT**  **(N=58)** | ***P*^*^** | **Before IPTW** | **After IPTW** |
|  |  |  |  | **RR/cOR**  **(95% CI)^†^** | **RR/cOR**  **(95% CI)^‡^** |
| mRS at 3 months**^\|\|^** |  |  |  |  |  |
| mRS 0-3, n (%) | 79 (44.4) | 20 (34.5) | .18 | 1.20 (0.82-1.77) | 1.27 (0.82-1.95) |
| mRS 0-2, n (%) | 52 (29.2) | 12 (20.7) | .20 | 1.28 (0.75-2.21) | 1.28 (0.71-2.29) |
| ordinal mRS, median (IQR) | 4 (2-5) | 5 (3-6) | .04 | 1.52 (0.86-2.70) | 1.74 (1.01-3.01) |
| Mortality at 3 months, n (%) | 34 (19.1) | 21 (36.2) | .01 | 0.58 (0.36-0.92) | 0.54 (0.34-0.85) |
| Symptomatic ICH, n (%) | 7 (3.9) | 3 (5.2) | .71 | NA | NA |
|  | **BAOCHE-eligible (N=61)** | | | | |
|  | **EVT**  **(N=36)** | **No EVT**  **(N=25)** | ***P*^*^** | **Before IPTW** | **After IPTW** |
|  |  |  |  | **RR/cOR**  **(95% CI)^§^** | **RR/cOR**  **(95% CI)** |
| mRS at 3 months**^\|\|^** |  |  |  |  |  |
| mRS 0-3, n (%) | 23 (63.9) | 13 (52.0) | .35 | 1.12 (0.74-1.70) | NA |
| mRS 0-2, n (%) | 15 (41.7) | 8 (32.0) | .44 | 1.23 (0.61-2.47) | NA |
| ordinal mRS, median (IQR) | 3 (1-5) | 3 (2-5) | .10 | 1.96 (0.77-4.95) | NA |
| Mortality at 3 months, n (%) | 5 (13.9) | 6 (24.0) | .33 | 0.64 (0.24-1.69) | NA |
| Symptomatic ICH, n (%) | 0 (0.0) | 0 (0.0) | NA | NA | NA |
|  | **Ineligible for both trials (N=695)** | | | | |
|  | **EVT**  **(N=304)** | **No EVT**  **(N=391)** | ***P*^*^** | **Before IPTW** | **After IPTW** |
|  |  |  |  | **RR/cOR**  **(95% CI)^†^** | **RR/cOR**  **(95% CI)^‡^** |
| mRS at 3 months^\|\|^ |  |  |  |  |  |
| mRS 0-3, n (%) | 120 (39.5) | 169 (43.2) | .32 | 1.25 (1.08-1.46) | 1.25 (1.07-1.47) |
| mRS 0-2, n (%) | 90 (29.6) | 133 (34.0) | .22 | 1.15 (0.95-1.40) | 1.15 (0.93-1.43) |
| ordinal mRS, median (IQR) | 4 (2-6) | 4 (2-6) | .43 | 1.48 (1.10-2.00) | 1.40 (1.06-1.86) |
| Mortality at 3 months, n (%) | 85 (28.0) | 113 (28.9) | .79 | 0.85 (0.67-1.07) | 0.79 (0.63-1.00) |
| Symptomatic ICH, n (%) | 6 (2.0) | 8 (2.0) | .95 | NA | 1.05 (0.35-3.16) |

Abbreviations: ICH, intracranial hemorrhage; IPTW, inverse probability of treatment weighting; IQR, interquartile range; mRS, modified Rankin scale; NA, not applicable; CI, confidence interval; cOR, common odds ratio; RR, risk ratio.

^†^Modified Poisson regression or ordinal logistic regression for lower mRS was applied adjusting for age, sex, diabetes, history of stroke or transient ischemic attack, initial stroke severity, pre stroke mRS, causative mechanism, intravenous thrombolysis, systolic blood pressure, transfer-in status, elapsed time from time last known well to arrival, and PC-ASPECTS.

^‡^Weighted modified Poisson regression or ordinal logistic regression with robust standard errors was applied, adjusting for variables showing imbalances after applying stabilized IPTW including age, hypertension, smoking, stroke or TIA, and causative mechanism.

**^§^**Modified Poisson regression or ordinal logistic regression for lower mRS was applied adjusting for age.

**Table S5. Association of EVT with clinical outcomes by trial eligibility and epoch**

|  | **ATTENTION-eligible (N=246)** | | **Ineligible for both trials (N=727)** | |
| --- | --- | --- | --- | --- |
|  | **After IPTW** | ***P* for interaction** | **After IPTW** | ***P* for interaction** |
|  | **RR/cOR (95% CI)**^*^ |  | **RR/cOR (95% CI)**^*^ |  |
| mRS 0-3 at 3 months | | | | |
| 2011-2014 | 0.82 (0.44-1.54) | .31 | 1.29 (0.91-1.83) | .57 |
| 2015-2018 | 1.23 (0.57-2.65) |  | 1.02 (0.75-1.38) |  |
| 2019-2021 | 2.27 (0.71-7.27) |  | 1.05 (0.74-1.47) |  |
| mRS 0-2 at 3 months | | | | |
| 2011-2014 | 0.56 (0.24-1.30) | .20 | 1.07 (0.69-1.67) | .76 |
| 2015-2018 | 1.33 (0.50-3.56) |  | 1.06 (0.74-1.52) |  |
| 2019-2021 | 2.83 (0.42-18.91) |  | 0.88 (0.57-1.35) |  |
| 3-month mRS, ordinal | | | | |
| 2011-2014 | 0.80 (0.33-1.94) | .12 | 1.44 (0.90-2.30) | .58 |
| 2015-2018 | 2.32 (0.96-5.60) |  | 1.13 (0.74-1.75) |  |
| 2019-2021 | 2.77 (1.06-7.20) |  | 1.01 (0.63-1.63) |  |
| Mortality at 3 months | | | | |
| 2011-2014 | 0.78 (0.35-1.72) | .42 | 0.69 (0.45-1.05) | .80 |
| 2015-2018 | 0.47 (0.21-1.01) |  | 0.83 (0.53-1.31) |  |
| 2019-2021 | 0.34 (0.12-0.97) |  | 0.83 (0.52-1.30) |  |

Abbreviations: IPTW, inverse probability of treatment weighting; mRS, modified Rankin scale; cOR, common odds ratio; RR, risk ratio.

^*^Weighted modified Poisson regression or ordinal logistic regression with robust standard errors was applied, adjusting for variables showing imbalances after applying stabilized IPTW.

**
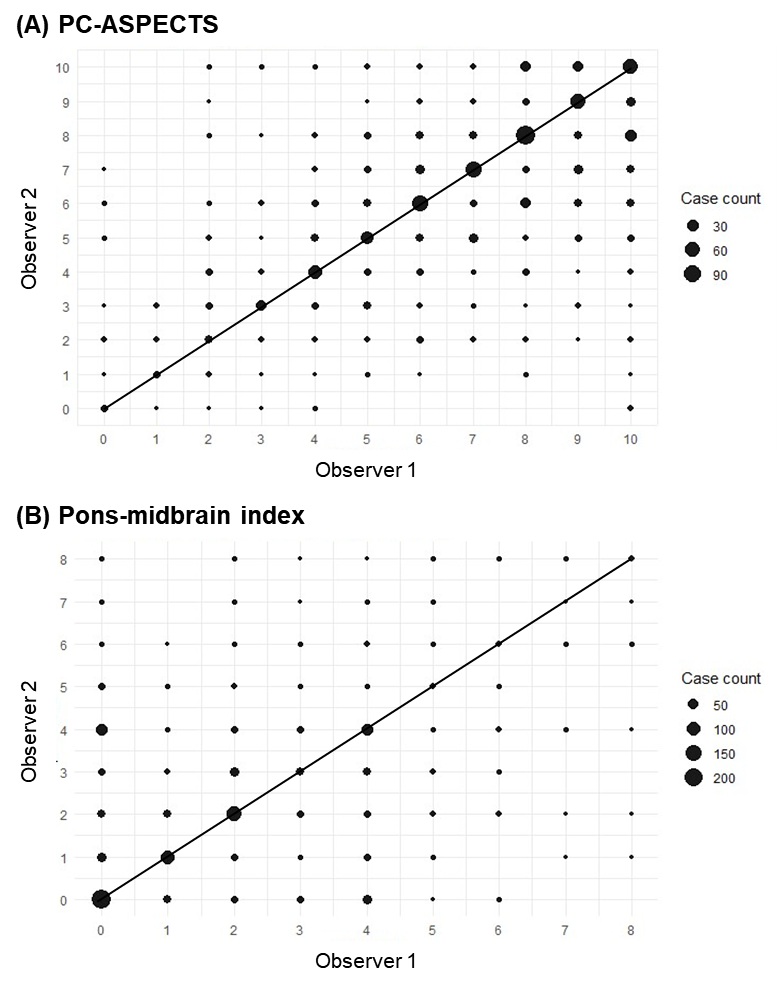
**

**Figure S1. Interobserver agreement for neuroimaging measures**

Weighted Cohen’s kappa values were calculated to assess interobserver agreement for PC-ASPECTS and the pons-midbrain index, based on paired ratings from two independent reviewers per patient.

Each case was evaluated by two randomly assigned raters selected from a pool of 18 vascular neurologists and neuroradiology specialists. All raters were blinded to clinical data. Kappa values were 0.46 for PC-ASPECTS and 0.50 for the pons-midbrain index, indicating moderate agreement.

Abbreviations: PC-ASPECTS, Posterior Circulation Acute Stroke Prognosis Early CT Score.


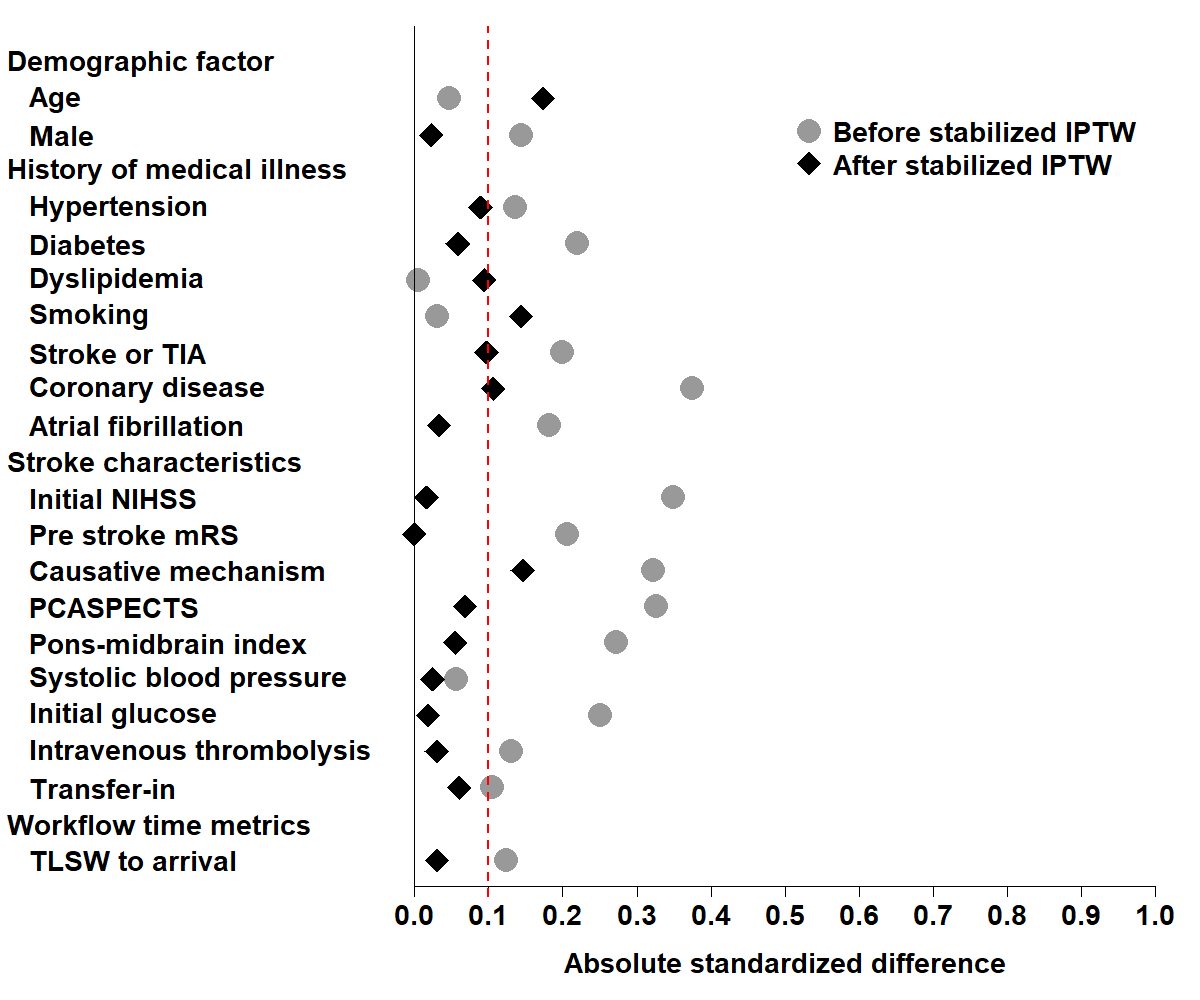


**Figure S2. Baseline characteristics before and after applying IPTW in patients eligible for ATTENTION trial**

Abbreviations: IPTW, inverse probability of treatment weighting; mRS, modified Rankin Scale; NIHSS, national institutes of health stroke scale; PCASPECTS, posterior circulation acute stroke prognosis early CT score; TIA, transient ischemic attack; TLSW, time last seen well.


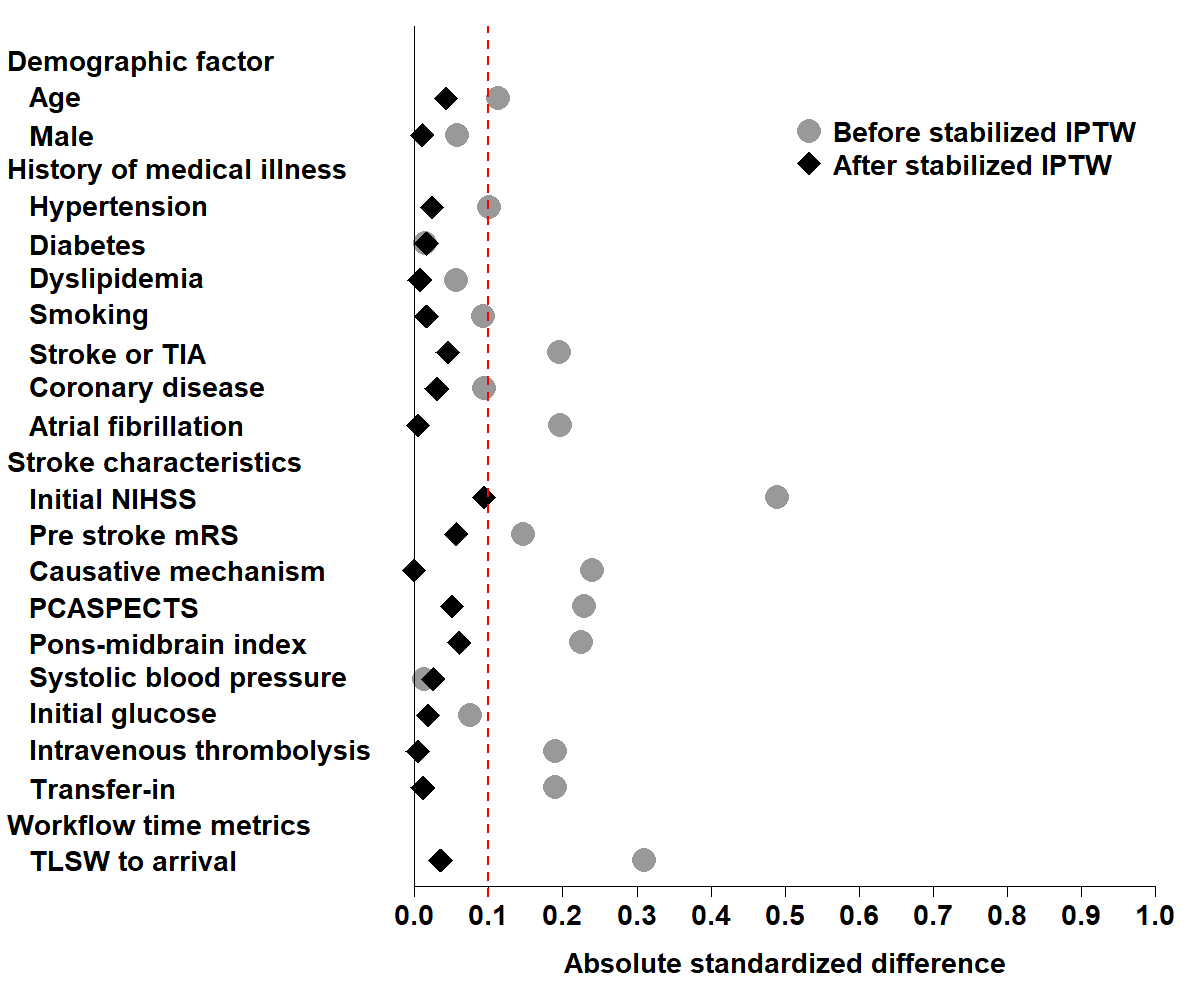


**Figure S3. Baseline characteristics before and after applying IPTW in patients ineligible for both trials**

Abbreviations: IPTW, inverse probability of treatment weighting; mRS, modified Rankin Scale; NIHSS, national institutes of health stroke scale; PCASPECTS, posterior circulation acute stroke prognosis early CT score; TIA, transient ischemic attack; TLSW, time last seen well.
